# Selective intraarterial hypothermia combined with mechanical thrombectomy for acute cerebral infarction based on microcatheter technology: A single-center, randomized, single-blind controlled study

**DOI:** 10.3389/fneur.2023.1039816

**Published:** 2023-02-16

**Authors:** Yue Wan, Hao Tian, Hui Wang, DaPeng Wang, HaiWei Jiang, Qi Fang

**Affiliations:** ^1^Department of Neurology, The First Affiliated Hospital of Suzhou University, Suzhou, Liaoning, China; ^2^Department of Neurology, Hubei Provincial Third People's Hospital, Zhongshan Hospital, Wuhan, Hubei, China

**Keywords:** infarction, endovascular therapy, hypothermia, controlled studies, oxidative stress, inflammatory response

## Abstract

**Objective:**

To investigate the safety and efficacy of selective intraarterial hypothermia combined with mechanical thrombectomy in the treatment of acute cerebral infarction based on microcatheter technology.

**Methods:**

A total of 142 patients with anterior circulation large vessel occlusion were randomly assigned to the hypothermic treatment group (test group) and the conventional treatment group (control group). National Institutes of Health Stroke Scale (NIHSS) scores, postoperative infarct volume, the 90-day good prognosis rate (modified Rankin Scale (mRS) score ≤ 2 points), and the mortality rate of the two groups were compared and analyzed. Blood specimens were collected from patients before and after treatment. Serum levels of superoxide dismutase (SOD), malondialdehyde (MDA), interleukin-6 (IL-6), IL-10, and RNA-binding motif protein 3 (RBM3) were measured.

**Results:**

The 7-day postoperative cerebral infarct volume [(63.7 ± 22.1) ml vs. (88.5 ± 20.8) ml] and NIHSS scores at postoperative Days 1, 7, and 14 [(6.8 ± 3.8) points vs. (8.2 ± 3.5) points; (2.6 ± 1.6) points vs. (4.0 ± 1.8) points; (2.0 ± 1.2) points vs. (3.5 ± 2.1) points] in the test group were significantly lower than those in the control group. The good prognosis rate at 90 days postoperatively (54.9 vs. 35.2%, *P* = 0.018) was significantly higher in the test group than in the control group. The 90-day mortality rate was not statistically significant (7.0 vs. 8.5%, *P* = 0.754). Immediately after surgery and 1 day after surgery, SOD, IL-10, and RBM3 levels in the test group were relatively higher than those in the control group, and the differences were statistically significant. Immediately after surgery and 1 day after surgery, MDA and IL-6 levels in the test group were relatively reduced compared with those in the control group, and the differences were statistically significant (*P* < 0.05). In the test group, RBM3 was positively correlated with SOD and IL-10.

**Conclusion:**

Mechanical thrombectomy combined with intraarterial cold saline perfusion is a safe and effective measure for the treatment of acute cerebral infarction. Postoperative NIHSS scores and infarct volumes were significantly improved with this strategy compared with simple mechanical thrombectomy, and the 90-day good prognosis rate was improved. The mechanism by which this treatment exerts its cerebral protective effect may be by inhibiting the transformation of the ischaemic penumbra of the infarct core area, scavenging some oxygen free radicals, reducing inflammatory injury to cells after acute infarction and ischaemia–reperfusion, and promoting RBM3 production in cells.

## Introduction

The results of several large randomized controlled clinical studies published in 2015 showed that stroke patients with anterior circulation large vessel occlusion should receive timely vascular recanalization therapy (1–5). Due to the rapid development of endovascular interventional therapy, a vascular recanalization rate of 66–94% can be achieved. However, only 46% of patients undergoing endovascular treatment have a good prognosis after 90 days, and ~15% of patients eventually die (6). Therefore, on the basis of intravascular interventional therapy to achieve vascular recanalization, we urgently need new ancillary therapeutic strategies to improve the prognosis of AIS patient. As early as 1987, Busto et al. found that reducing the brain temperature by only a few degrees during ischaemia can produce a significant neuroprotective effect (7). In recent years, therapeutic hypothermia has been considered the most effective neuroprotective strategy. Many clinical studies of acute cerebral infarction have also shown that mild hypothermia (33–35°C) treatment can increase the tolerance of brain cells to ischemic injury, and after vascular recanalization, hypothermia can continue to play a protective role in neurological function (8, 9). However, although traditional therapeutic hypothermia has been demonstrated to be effective for ischaemic stroke in animal experiments, its application in clinical practice has resulted in a series of complications, especially pneumonia. Therefore, therapeutic hypothermia is not recommended as a routine treatment method in current clinical guidelines for acute stroke (10–12). At present, a novel hypothermia strategy has emerged; that is, selective brain hypothermia that does not need to reduce core body temperature. Thus, in theory, many serious adverse effects caused by systemic hypothermia can be avoided. Recently, Chen and Wu successively published studies confirming that short-duration injecting cold saline into the microcatheter to treat acute cerebral infarction during mechanical thrombectomy is feasible and safe (13, 14). However, randomized control trials (RCTs) investigating the safety and efficacy of selective intraarterial hypothermia combined with mechanical thrombectomy based on microcatheter technology in the treatment of acute cerebral infarction are currently lacking.

Cold-induced proteins, also known as cold shock proteins, are associated with the neuroprotective effect of hypothermia (15, 16). Hypothermia inhibits cell metabolism and most protein synthesis in the body, but promotes cold shock protein synthesis (17–19). Previous basic studies have shown that cold-induced RNA-binding motif protein 3 (RBM3) plays a key role in organ protection during hypothermia treatment (16, 20, 21). To date, many data on RBM3 have been obtained in cell culture or animal models. RBM3 can be detected and regulated in human neurons *in vitro*, but few studies are available on the detection and regulation of RBM3 in patients' blood. At the same time, although therapeutic hypothermia is considered to have considerable potential in the treatment of ischaemic stroke, the specific role of RBM3 in treatment is still unclear.

Oxidative stress is an important mechanism of ischaemic stroke cell injury (22, 23). In the acute phase of ischaemia, mitochondrial dysfunction and increased production of reactive oxygen species (ROS) occur (24). ROS can oxidize unsaturated fatty acids on cell membranes, and the final product of the reaction is malondialdehyde (MDA) (25, 26). Superoxide dismutase (SOD) is an important antioxidant enzyme in the body and an important free radical scavenger. MDA and SOD levels can indirectly reflect the body's oxidative and antioxidative abilities (27). The oxidative stress response also leads to the increased release of proinflammatory cytokines (28). The main proinflammatory cytokines include tumor necrosis factor-α (TNF-α), interleukin (IL)-1β, IL-6, and chemokines. Anti-inflammatory factors such as IL-10 and transforming growth factor (TGF) -β have neuroprotective effects.

Therefore, based on the above research background, we conducted a single-center, prospective, randomized, single-blind controlled study to investigate the safety and efficacy of selective intraarterial cooling combined with mechanical thrombectomy in the treatment of acute cerebral infarction based on microcatheter technology. At the same time, MDA, SOD, IL-6, IL-10, and RBM3 levels in the blood of patients were measured and compared.

## Methods

### Study design and research subjects

This study used a single-center, randomized, single-blind, controlled study. Previous studies has shown that the rates of 90-day functional independence (mRS scores of 0–2) in patients selected for mechanical thrombectomy by CTP are 41.3–45%. Wu et al. conducted a prospective non-randomized cohort study of 113 consecutive patients to investigate the safety and efficacy of a short-duration IA-SCI combined with MT in patients with large vessel occlusion-induced acute ischemic stroke. They found that an aOR of a favorable outcome (mRS, 0–2) was 1.9. The Wu study's hypothermia therapy lasted 15 min. According to preliminary studies, the advantages of hypothermia treatment will increase with longer time. By improving the mode of hypothermia, the duration of hypothermia in our study was extended to 35 min. Thus, better therapeutic results can be expected in our study. The estimated odds ratio (OR) in our study was 2.8. The sample size was calculated based on PASS software 11.0. The test level was set to α = 0.05, the power level 1-β was set to 80%, and the intergroup ratio was predicted to be 1:1. Considering the possibility of loss to follow-up, the calculation result was that at least 62 patients needed for each group, with a total of at least 124 patients. Subjects provided written informed consent to participate in the study. This study was approved by the Medical Ethics Committee of the hospital.

The inclusion criteria were as follows: (1) age >18 years; (2) within 24 h from onset to randomization, although the DAWN or DEFUSE 3 study criteria were used when the this time exceeded 6–24 h. (3) an NIHSS score ≥6; (4) cerebral hemorrhage excluded by cranial CT; (5) a preonset modified Rankin Scale (mRS) score ≤ 1; (6) stroke with anterior circulation large vascular occlusion confirmed by CT angiography (CTA)/digital subtraction angiography (DSA); (note: the anterior large vascular circulation was defined as the M1 and M2 segments of the internal carotid artery (ICA) and the middle cerebral artery (MCA), including the extracranial and intracranial segments); and (7) signed written informed consent forms.

Exclusion criteria: (1) genetic or acquired haemorrhagic constitution, anticoagulant factor deficiency, or oral anticoagulant drug use, and an international normalized ratio (INR)>3; platelet count < 40 × 10^9^/L, activated partial thromboplastin time (aPTT) > 50 s; (2) systolic blood pressure > 220 or diastolic blood pressure >110 mm Hg; (3) blood glucose < 2.8 mmol/L (50 mg/dl) or > 22.2 mmol/L (400 mg/dl); and (4) life expectancy < 1 year due to any late-stage disease; (5) allergy to contrast agents; (6) a history of alteplase or urokinase intravenous thrombolytic therapy; (7) previous cardiovascular and cerebrovascular interventional surgery or other major surgery within 48 h; (8) severe liver dysfunction, alanine aminotransferase (ALT) >3 times the upper limit of normal, or aspartate transaminase (AST) >3 times the upper limit of normal or chronic haemodialysis and severe renal insufficiency (glomerular filtration rate < 30 ml/min or serum creatinine >220 μmol/L (2.5 mg/dl); (9) pregnancy or lactation; (10) participation in other clinical trials that may have an impact on this study; (11) intracranial infection, intracranial aneurysm, o arteriovenous malformation; (12) myocardial infarction within 30 days; (13) ejection fraction < 40%, insufficiency of vital organs such as the heart and lungs; (14) current severe alcohol dependence or drug abuse; (15) Alzheimer's disease or mental illness affecting follow-up reliability; and (16) an expectation that the follow-up would not be completed.

### Treatment method

In the catheterization laboratory, the patient was placed in the supine position, followed by oxygen administration, electrocardiogram (ECG), blood pressure, and oxygen saturation monitoring, urinary catheterization, and general anesthesia with endotracheal intubation. Routine bilateral disinfection and draping of the inguinal area was performed. The Seldinger technique was used to place an 8F vascular sheath after femoral artery puncture, and Door Puncture Time (DPT) was recorded, followed by systemic heparinization. Under the guidance of an angiographic guidewire, a 5F “pigtail” catheter and a “single bend” catheter were placed in the aortic arch, bilateral carotid, and vertebral arteries for angiography to determine the vascular occlusion location, vascular alignment, and blood flow compensation in the area of occlusion. The 8F guiding catheter or a long sheath was placed in the common carotid artery or ICA on the lesion side with the help of the angiography guidewire and the multifunctional catheter, and then the middle catheter was placed at the proximal end of the arterial occlusion. A shaped Synchro microguidewire (260 cm, Stryker), together with a Rebar microcatheter (18/27, EV3), were carefully passed through the occlusion site. After withdrawal of the microguidewire, smoke in the microcatheter indicated patency of the distal vessel, and the position of the microcatheter was appropriately adjusted (to facilitate complete coverage of the thrombus after stent release). In the hypothermic treatment group, 4°C normal saline was perfused into the microcatheter at 15 ml/min for 5 min, and then the occluded blood vessels were opened using the SOLUMBAR or SWIM technique. If necessary, balloon dilation and stent placement were combined to open the blood flow. After successful revascularization, the guide catheter or the long sheath was perfused with 4°C normal saline at 22 ml/min, and perfusion was continued for 10 min. After 10-min intervals, 4°C normal saline was continuously perfused for 10 min. No abnormalities requiring termination of the procedure were observed on repeated angiography. The control group received a conventional mechanical thrombectomy with a stent retriever to recanalize the occluded vessel as soon as possible.

### Observation indicators

Major safety endpoint events:

The incidence of symptomatic intracranial hemorrhage at 24 h after treatment (using the Heidelber criteria) and the 90-day mortality rate.

Secondary safety endpoint events:

(1) Angiography performed 10 min after cold saline perfusion showing the incidence of vasospasm;
(2) The incidence of abnormal haematocrit (HCT) at 3 h after surgery;
(3) Abnormal blood coagulation 3 h after surgery;
(4) The incidence of pneumonia within 7 days after surgery;
(5) The incidence of urinary tract infection within 7 days after surgery.

Primary efficacy endpoint events:

The percentage of patients with a good prognosis at 90 days (mRS score 0–2 points).

Secondary efficacy endpoint events:

(1) The difference in the final infarct volume (FIV) between the two groups;
(2) Changes in NIHSS scores at 1 day after surgery;
(3) Changes in NIHSS scores at 7 days after surgery;
(4) Changes in NIHSS scores at 14 days after surgery;
(5) Changes in RBM3 levels before surgery, immediately after surgery, and 1 day after surgery;
(6) Changes in SOD and MDA levels before surgery, immediately after surgery, and 1 day after surgery;
(7) Changes in IL-6 and IL-10 levels before surgery, immediately after surgery, and 1 day after surgery.

### Statistical analysis methods

Statistical analysis was performed on various baseline indicators (such as medical history) and demographic characteristic indicators. Intention-to-treat analysis was selected for the statistical analysis of experimental efficacy, and the dataset that met the protocol (i.e., the statistical analysis was performed based on the data of all patients who met the criteria of the experimental protocol) was used as the reference. The measurement data were statistically described using the mean, median, standard deviation, maximum, minimum, and 25 and 75% quantiles, and the group *t*-test or Mann–Whitney *U*-test was used to compare two groups. Count data or ranked data were statistically described using frequencies. The χ^2^ test or Fisher's exact probability method was used to compare the count data between the two groups, and the Mann-Whitney U test was used to compare the ranked data between the two groups.

## Results

### Patients

**Figure 1:**
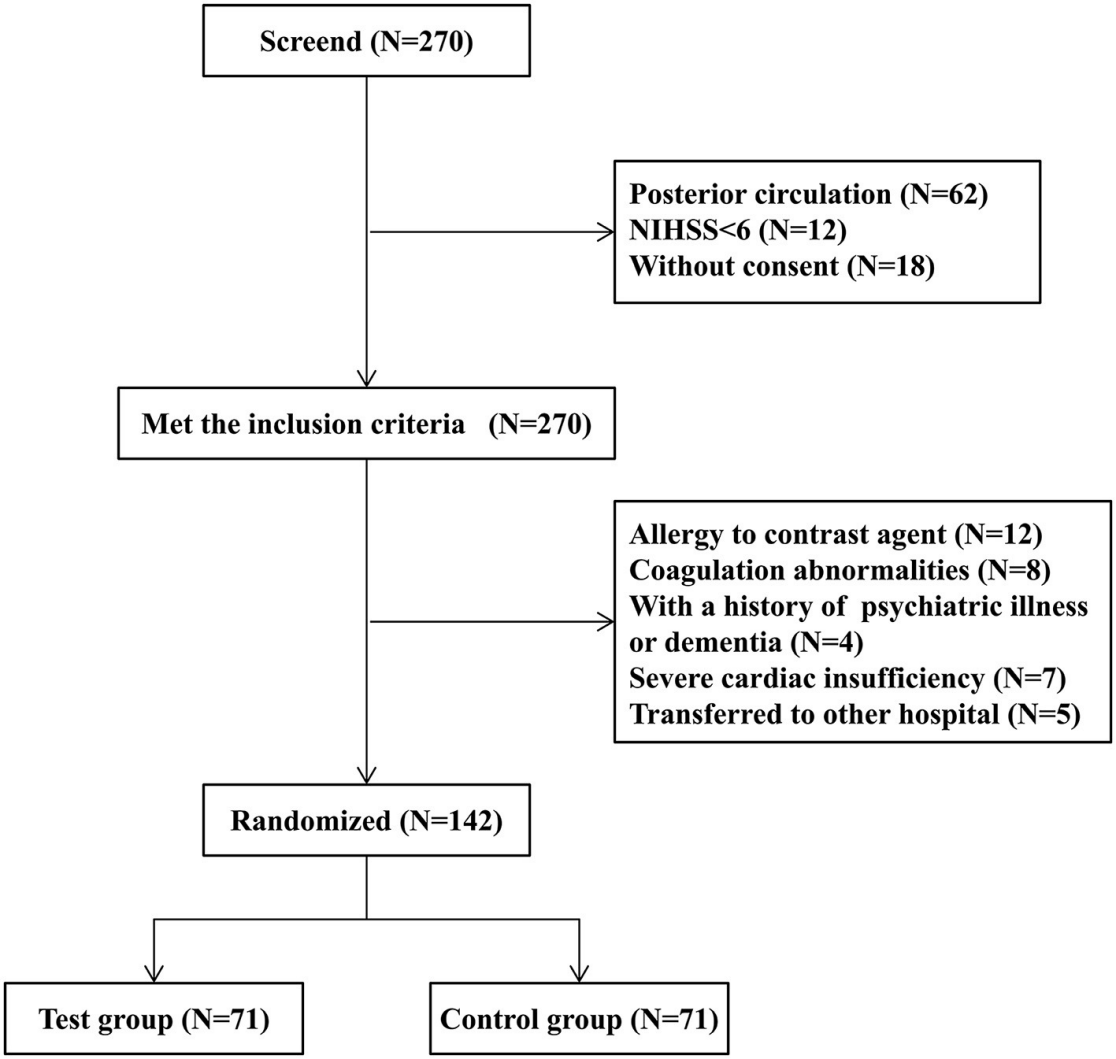
The study flow diagram.

Between September 2019 and March 2022, 270 patients were screened, and 178 patients met the inclusion criteria. Among them, 36 patients were excluded due to reasons such as a history of contrast agent allergy, abnormal coagulation function, mental illness, poor cardiac function, and hospital transfer during the study. A total of 142 patients were actually randomized to the groups. Figure 1 summarizes the process of patient recruitment and participation. The average age of the 71 patients in the test group was 73.4 years, 44 (62.0%) of whom were males, while 50 (70.4%) had hypertension, 18 (25.3%) had diabetes, 25 (35.2%) had coronary heart disease, and 36 (50.7%) had a smoking history. The average time from onset to femoral artery puncture was 258 min, the average time from onset to vascular recanalization was 343 min, the average NIHSS score at admission was 15, the average IC area volume was 58.5 ml, and 35 patients (49.3%) had poor collateral circulation. The average age of the 71 patients in the control group was 72.8 years, 40 (56.3%) of whom were males, while 52 (73.2%) had hypertension, 16 (22.5%) had diabetes, 28 (39.4%) had coronary heart disease, and 39 (54.9%) had smoking history. The average time from onset to femoral artery puncture was 279 min, the average time from onset to vascular recanalization was 361 min, the average NIHSS score at admission was 16, the average IC area volume was 60.4 ml, and 36 patients (50.7%) had a poor collateral circulation. No statistically significant difference in the general clinical data was found between the two groups (Table 1).

**Table 1:** Baseline clinical data of the patients.

**Variable**	**Test group (*n* = 71)**	**Control group (*n* = 71)**	***P***
Age, year	73.4 ± 12.6	72.8 ± 13.6	0.621
Male	44 (62.0%)	40 (56.3%)	0.495
Hypertension	50 (70.4%)	52 (73.2%)	0.709
Diabetes	18 (25.3%)	16 (22.5%)	0.694
Coronary heart disease	25 (35.2%)	28 (39.4%)	0.603
Smoke	36 (50.7%)	39 (54.9%)	0.614
Time from onset to puncture, min	258 ± 71	279 ± 60	0.485
Time from onset to recanalization, min	343 ± 80	361 ± 92	0.603
NIHSS score	15 ± 7	16 ± 8	0.712
Infarct core volume, ml	58.5 ± 25	60.4 ± 31.8	0.814
Poor collateral circulation	35 (49.3%)	36 (50.7%)	0.867

### Efficacy

**Figure 2:**
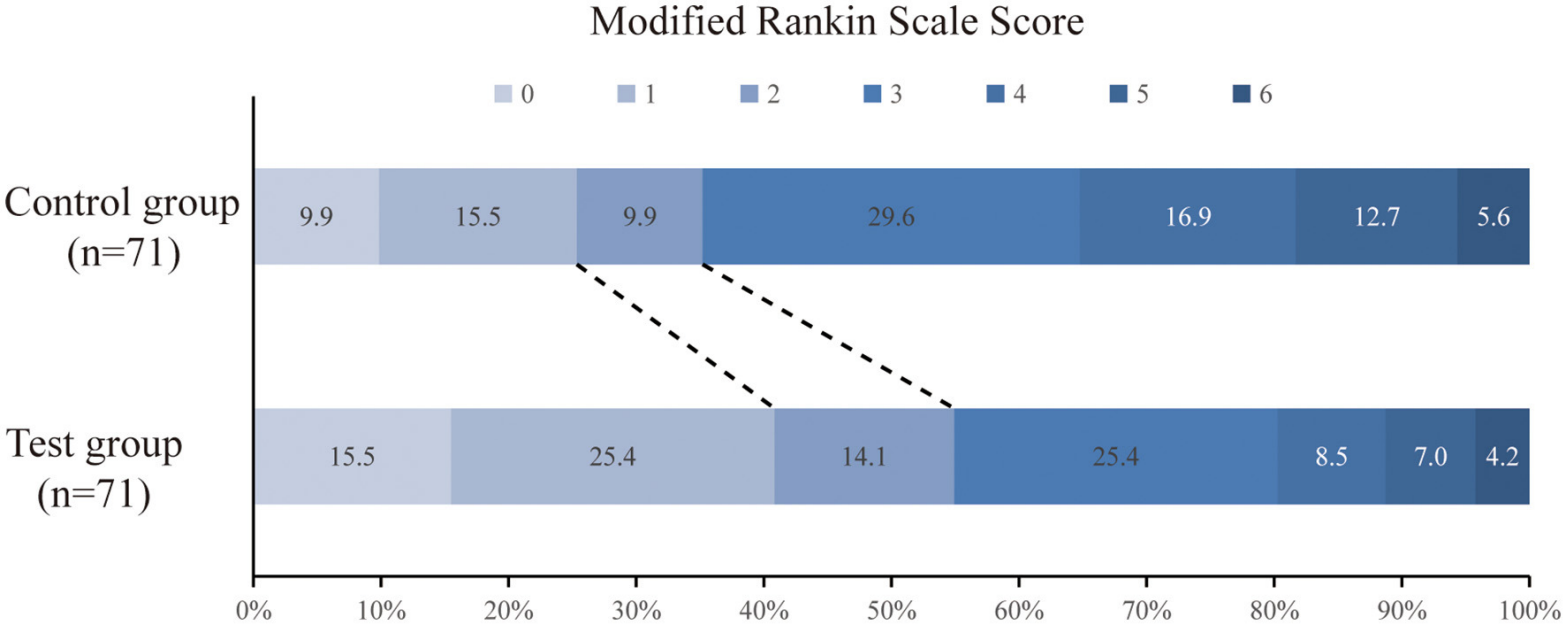
Distribution of scores on the modified Rankin Scale at 90 d.

**Figure 3:**
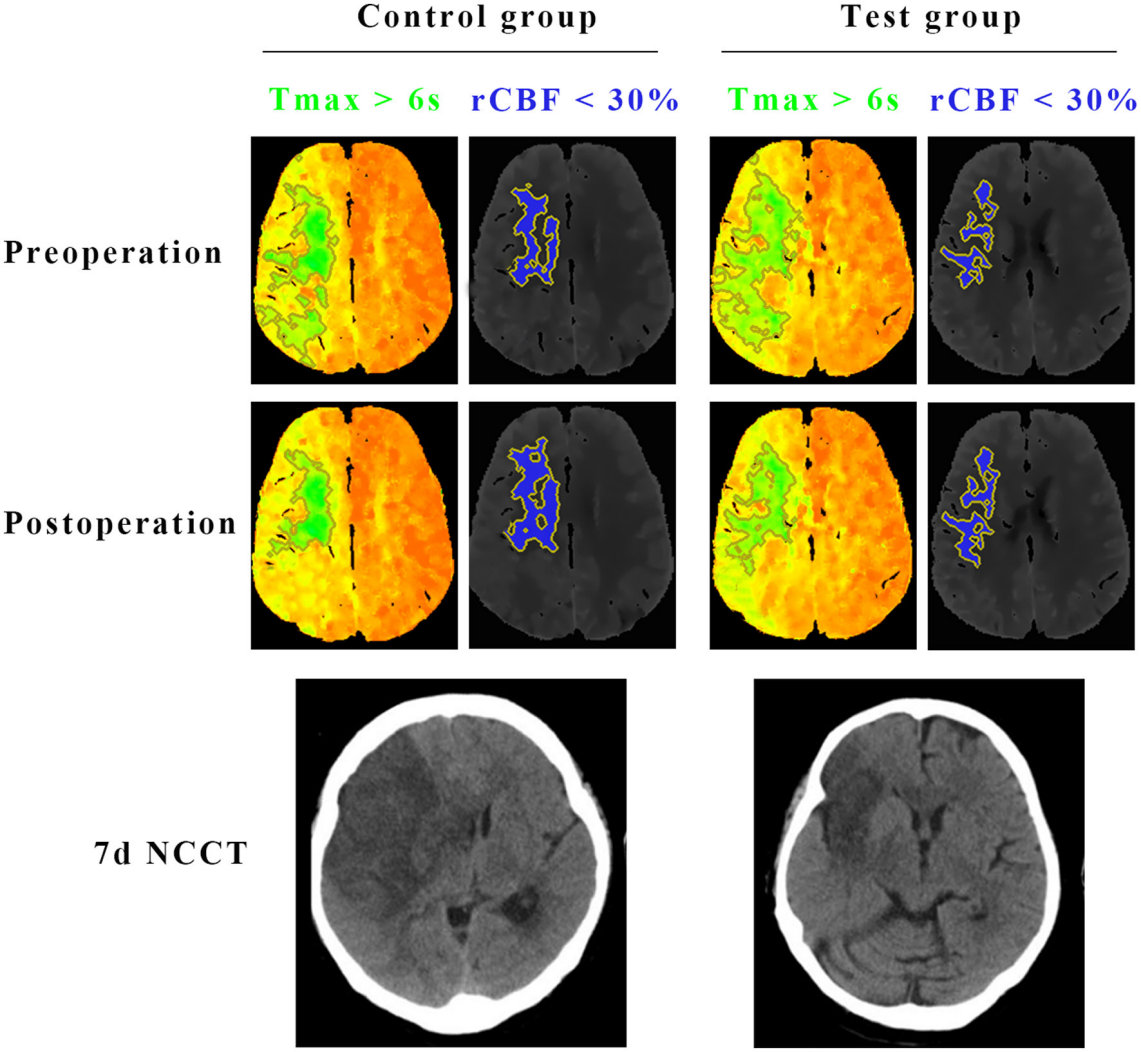
Comparisons of the infarct core (rCBF < 30%) and hypoperfused tissue (Tmax >6 s) on CTP, and final infarct volume on CT between test group and control group. Preoperative CTP of a representative patient in the test group showed 93 ml of Tmax >6 s (green) and 26.5 ml of < 30% rCBF (blue). The CTP demonstrated a penumbral pattern (infarct core < 70 mL, penumbra >15 mL, mismatch >1.8). At 7 days after the surgery, CTP of the same patient in the test group showed 35.6 ml of Tmax >6 s (green) and 29.5 ml of < 30% rCBF (blue). Final infarct volume on CT of the patient in the test group was 43.4 ml. Preoperative CTP of the other representative patient in the control group showed 91.2 ml of Tmax >6 s (green) and 30.2 ml of < 30% rCBF (blue). The CTP demonstrated a penumbral pattern as well (infarct core < 70 mL, penumbra >15 mL, mismatch >1.8). At 7 days after the surgery, CTP of the same patient in the control group showed 36.7 ml of Tmax >6 s (green) and 32.4 ml of < 30% rCBF (blue). Final infarct volume on CT of the patient in the control group was 85.3 ml.

The primary efficacy was determined as the percentage of patients with a good prognosis at 90 days (mRS score 0–2 points). At 90 days after surgery, the rate of a good prognosis in the test group was significantly higher than that in the control group (54.9 vs. 35.2%, *P* = 0.018). The percentage of patients with severe disability (mRS score ≥4) in the test group was lower than that in the control group (19.7 vs. 35.2%, *P* = 0.039). The detailed mRS distribution of the two groups is shown in Figure 2. The preoperative IC area volumes in the test group and the control group were 58.5 ± 25 ml and 60.4 ± 31.8 ml, respectively, with no significant difference between the two groups (*P* = 0.814). CTP was re-examined 7 days after surgery. The IC area volumes in the test group and the control group were 63.7 ± 22.1 ml, and 88.5 ± 20.8 ml, respectively, with a significant difference between the two groups (*P* = 0.031) (Figure 3). Compared with that in the control group, the infarct volume in the test group was reduced by 28%. Compared with those in the control group, the NIHSS scores in the test group were significantly reduced at 1 d, 7 d, and 14 d after surgery, with *P*-values of 0.035, 0.026, and 0.018, respectively, and the results were all statistically significant (*P* < 0.05) (Table 2).

**Table 2:** The clinical indexes of the two groups were compared.

**Variable**	**Test group (*n* = 71)**	**Control group (*n* = 71)**	***P***
Final infarct volume	63.7 ± 22.1	88.5 ± 20.8	0.031
**NIHSS score**			
Postoperative 1d	6.8 ± 3.8	8.2 ± 3.5	0.035
Postoperative 7d	2.6 ± 1.6	4.0 ± 1.8	0.026
Postoperative 14d	2.0 ± 1.2	3.5 ± 2.1	0.018
mRS 0–2	39 (54.9%)	25 (35.2%)	0.018
mRS ≥4	14 (19.7%)	25 (35.2%)	0.039

**Figure 4:**
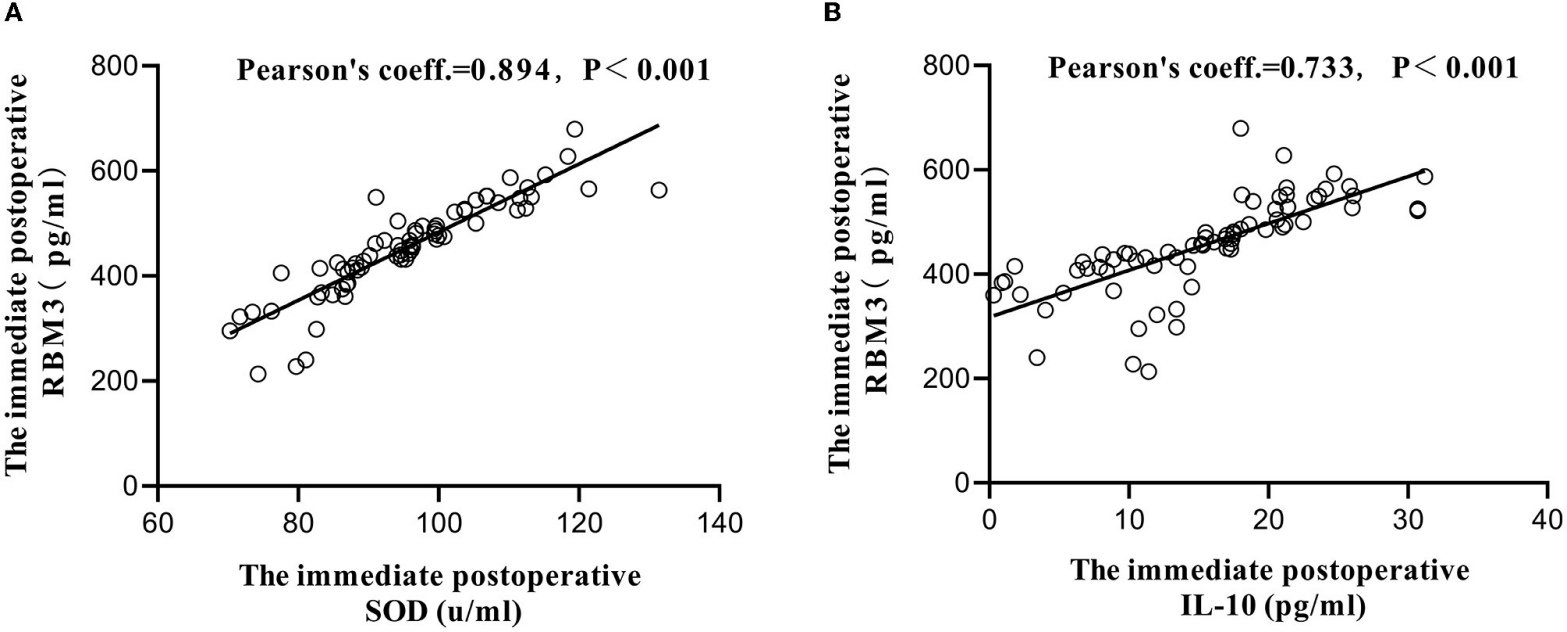
The relationship between RBM3 and SOD **(A)**, IL-10 **(B)** immediately after operation.

Before surgery, no significant difference in serum SOD, MDA, RBM3, IL-6, or IL-10 levels were identified between the two groups (*P* > 0.05). Compared with those in the control group, the SOD, IL-10, and RBM3 levels in the test group were increased immediately after surgery and at 1 day after surgery, and the results were significantly different (*P* < 0.05). MDA and IL-6 were relatively reduced, with significant differences (Table 3). Correlation analysis was performed on RBM3 and SOD levels immediately after surgery in the test group. The calculated Pearson correlation coefficient was 0.894, and the *P*-value was < 0.001, showing a positive correlation between the two. Correlation analysis was performed between RBM3 and IL-10 levels immediately after surgery in the test group. The calculated Pearson correlation coefficient was 0.733, and the *P*-value was < 0.001, also showing a positive correlation between the two. This finding indicates that RBM3 is closely related to SOD and IL-10 (Figure 4).

**Table 3:** Comparison of efficacy laboratory indexes between the two groups.

**Group**	**Indicators**	**Preoperative**	**Postoperative**	**Postoperative 1d**
Test group	RBM3	356.8 ± 150.4	454.4 ± 90.6^*#^	410.8 ± 20.1^*#^
	SOD	73.3 ± 17.3a	95.6 ± 12.5^*#^	107.4 ± 9.9b^#^
	MDA	10.8 ± 0.5	8.3 ± 0.7^*#^	5.4 ± 0.3b^#^
	IL-6	40.2 ± 19.3	45.6 ± 22.4^*#^	44.2 ± 25.2^*#^
	IL-10	11.9 ± 6.9	15.2 ± 7.4^*#^	17.4 ± 6.8^*#^
Control group	RBM3	364.2 ± 161.7	378 ± 140.5	350.4 ± 150.9
	SOD	72.4 ± 18.4	76.5 ± 19.5^*^	88.3 ± 18.6^*^
	MDA	10.8 ± 0.9	9.5 ± 0.6^*^	8.1 ± 0.4^*^
	IL-6	39.8 ± 21.4	49.7 ± 23.6^*^	53.1 ± 28.6^*^
	IL-10	10.8 ± 7.1	13.1 ± 6.6^*^	12.9 ± 7.6^*^

^*^On behalf of *P* < 0.05 vs. Preoperative, ^#^ on behalf of *P* < 0.05 vs. Control group.

### Safety

Rectal temperature was monitored every 5 min during the operation. The average minimum rectal temperature after the first infusion of cold saline in the test group was 36.5 ± 0.7°C. The average minimum temperature of the second infusion of cold saline was 36.6 ± 0.5°C. The average minimum temperature of the third infusion of cold saline was 36.6 ± 0.7°C. Repeated measures analysis of variance was used to compare the two groups. No significant difference in rectal temperature was noted between the two groups during the entire process (*P* = 0.493).

Symptomatic intracranial hemorrhage was observed in 11 patients (15.5%) in the test group and in 13 patients (18.3%) in the control group (*P* >0.05). Intracranial vasospasm occurred in 2 patients (2.8%) in the test group and in 1 patient (1.4%) in the control group (*P*> 0.05). Five patients (7.0%) died in the test group and 6 (8.5%) in the control group. Three (4.2%) patients in the test group had abnormal HCT at 3 h after surgery, and one (1.4%) in the control group had abnormal HCT (*P*-value >0.05). Two patients (2.8%) in the test group and 3 (4.2%) patients in the control group had abnormal coagulation function (*P* > 0.05). Pneumonia occurred in 17 patients (23.9%) in the test group and in 15 patients (21.1%) in the control group (*P* > 0.05). Fifteen (21.1%) patients in the experimental group and 18 (25.4%) patients in the control group had urinary tract infections (*P*>0.05) (Table 4).

**Table 4:** Evaluation of safety.

**Variable**	**Test group (*n* = 71)**	**Control group (*n* = 71)**	***P***
Symptomatic intracranial hemorrhage	11 (15.5%)	13 (18.3%)	0.654
Death	5 (7.0%)	6 (8.5%)	0.754
Intracranial vasospasm	2 (2.8%)	1 (1.4%)	1
HCT abnormal	3 (4.2%)	1 (1.4%)	0.62
Coagulation abnormalities	2 (2.8%)	3 (4.2%)	1
Pneumonia	17 (23.9%)	15 (21.1%)	0.688
Urinary system infection	15 (21.1%)	18 (25.4%)	0.551

## Discussion

In this randomized controlled study, we found that selective intraarterial hypothermia combined with intravascular intervention for the treatment of patients with acute cerebral infarction based on microcatheter technology is safe. By monitoring rectal temperature, the core body temperature did not change significantly in the hypothermia treatment group. While traditional systemic hypothermia technology exerts its protective effect on neurological function, patients may experience a series of side effects, such as cardiovascular dysfunction, chills, immunosuppression, and impaired coagulation function (11, 29). Selective intraarterial hypothermia not only lowers the temperature quickly but also has fewer systemic side effects (15, 30–33). In this study, the biochemical and imaging results confirmed that although we administered a total of 515 ml of cold saline infusion, which was larger than the amount of 350 ml used by Chen and Wu, no significant abnormalities in HCT or coagulation factors were found. Postoperative angiography re-examination did not show an increase in the percentage of vasospasm in the hypothermia group (13, 14). Compared with those in the control group, no differences in the risk of bleeding and 90-day mortality were found in the hypothermia treatment group.

By comparing the IC volumes of the two groups at 7 days after surgery, we found that hypothermia treatment reduced the IC volume by 28%. Clearly, the reduced IC volume is achieved by saving the ischemic penumbra and avoiding transformation of this area into the irreversible IC area. In this study, the percentage of patients with a good clinical prognosis after 90 days in the hypothermia group was higher than that in the control group. In a previous study by Wu, the percentages of patients with a good prognosis were 51.1% in the hypothermia treatment group and 41.2% in the control group. Although the hypothermia treatment group showed an advantage, the difference was not statistically significant. Wu suggested that this result is because of a high percentage of poor collateral circulation in the preoperative hypothermia group, which weakened the advantage of the hypothermia treatment group (14). The patients enrolled in Wu's study were under 80 years of age and were enrolled within 6 h of onset, while the patients enrolled in this study were not limited to an age under 80 years. Furthermore, by using the CTP technique, patients with large vascular occlusion at 6–24 h of onset were screened and included in this study, resulting in a larger sample size than in Wu's study. The hypothermia treatment time in the study by Chen and Wu was 15 min (13, 14). Evidence from basic research shows that a longer duration of hypothermia treatment will result in greater benefits. Intraarterial infusion of cold saline will increase the hypothermia time, which will increase the amount of saline perfusion, which may lead to a series of side effects, such as electrolyte imbalance and blood dilution. To overcome this problem, Ji changed the continuous perfusion mode to the intermittent multiple perfusion mode by adjusting the perfusion regimen. Intermittent perfusion was found to be safe and feasible, not only prolonging the treatment time, but also contributing to the reduction in cerebral infarct volume in rats (34). Inspired by this finding, this study was improved on the basis of Chen and Wu's study. Only 165 ml of fluid was added, and the 15-min hypothermia treatment time was extended to 35 min. Finally, no significant difference in safety was observed between the two groups, and the hypothermia group had significant advantages. The overall efficacy significantly differed.

When acute cerebral infarction occurs, ischaemia causes a decrease in oxygen supply and a lack of ATP, and the intracellular and extracellular ion gradient Na^+^-K^−^ pumps will fail, causing calcium ion influx (35). Increased intracellular Ca^2+^, Na+, and ADP lead to mitochondrial dysfunction and increased (ROS) (36). ROS include superoxide anion (O-), hydroxyl radical (OH), and peroxide. SOD is an important antioxidant enzyme in the cell that can catalyze O_2_ and H_2_O_2_ generation from superoxide anion free radicals such that the intracellular superoxide anion free radicals are present at a harmless level. When brain tissue is deprived of blood and oxygen, SOD and other important antioxidant enzymes will also be damaged, and the increased ROS will consume a large amount of SOD. The free radical scavenging ability is reduced, and excessive accumulation in the body occurs. Excess oxygen free radicals and unsaturated fatty acids in brain tissue undergo lipid peroxidation to form lipid peroxides, which accumulate in the body and produce toxic effects on the human body. MDA is the final product of lipid peroxidation. Therefore, the level of SOD activity indirectly reflects the body's ability to scavenge free radicals, while MDA reflects the severity of the body's cells being attacked by free radicals (37). The results of this study showed that the SOD level in the test group was significantly higher than that in the control group immediately after hypothermia treatment and at 1 day after surgery. The MDA level in the test group immediately after surgery was lower than that in the control group, and MDA level at 1 day after surgery was significantly lower than that in the control group. The difference in MDA tended to be significant over time. These results suggest that selective intraarterial hypothermia combined with intravascular interventional therapy based on microcatheter technology can better alleviate oxidative stress, which is an important mechanism for hypothermia treatment to reduce the disability rate and improve the long-term prognosis of patients with cerebral infarction.

In ischaemic stroke, the inflammatory response plays the role of a double-edged sword, with both beneficial and harmful aspects. In fact, after stroke, the activation of microglia, astrocytes, and endothelial cells has a neuroprotective effect and can promote nerve regeneration and recovery. However, these cells recruit immune cells that express inflammatory mediators, resulting in disruption of the blood-brain barrier (BBB), which in turn leads to neuronal death, cerebral oedema, and haemorrhagic transformation (38). IL-6 is an important proinflammatory cytokine that can promote the inflammatory response of damaged cells in ischaemic stroke. It can be detected in the brain tissue, cerebrospinal fluid, and blood of patients with acute cerebral infarction and is associated with expansion of cerebral infarction (39). IL-10 is an anti-inflammatory factor. Previous studies have shown that it can promote tissue repair. In particular, IL-10 can reduce the toxic effect of excitatory amino acids on neurons after cerebral ischaemia (40–42). This study confirmed that hypothermia can inhibit IL-6, increase IL-10, and play a protective role in the brain.

Basic studies have confirmed that RBM3 plays a key role in hypothermia-induced neuroprotection, but research data on RBM3 detection from clinical studies are limited (17, 43–46). Our study found that RBM3 was elevated in the test group and was associated with a good prognosis, which provided convincing evidence for RBM3 as a novel therapeutic target in ischaemic stroke. Our study also found that RBM3 was positively correlated with IL-10 and SOD. This association may be the main mechanism by which RBM3 exerts its neuroprotective effect. Of course, the exact regulatory pathways between RBM3, IL-10, and SOD molecules require further study.

There are limitations in our study. Firstly, during the study, we monitored rectal temperature, but not brain parenchymal temperature. Currently, appropriate devices for direct monitoring of brain temperature in patients with acute cerebral infarction are lacking. Such a device needs to meet several requirements. First, the device must be non-invasive. In previous basic studies, probes were used to measure brain temperature, but they are not clinically applicable because they aggravate the risk of nerve cell damage and bleeding in patients. Second, the device for monitoring brain temperature must be installed easily and quickly. “Time is brain,” and the device should not delay the time to vascular patency due to the need to monitor brain temperature. Third, intraoperative real-time monitoring is needed. Regretfully, at present, there is no monitoring brain temperature device that meet the above requirements at the same time. Secondly, selective brain cooling through IA route do not qualify all patients with stroke. Although our study confirmed that this invasive cooling method is feasible and safe, not all patients need to afford a risk of procedure related complications. Lastly, there are no specialized cooling catheters in the market. In our study, we used routing catheters which are designed for mechanical thrombectomy, Thus, the thermal insulation performance of the catheters could affect the efficiency of selective intraarterial hypothermia. However, it is foreseeable that specialized medical devices for selective intraarterial hypothermia will be available in the near future with advancements in science and technology.

## Conclusion

Selective intraarterial hypothermia combined with mechanical thrombectomy based on microcatheter technology is safe and effective for the treatment of acute cerebral infarction. It inhibits transformation of the ischaemic penumbra to the infarct core area to some extent, scavenges some oxygen radicals, reduces cellular inflammatory damage after acute infarction and ischemia-reperfusion, and promotes cellular production of RBM3, which ultimately improves the functional prognosis of patients after 90 days.

## Data availability statement

The raw data supporting the conclusions of this article will be made available by the authors, without undue reservation.

## Ethics statement

The studies involving human participants were reviewed and approved by Ethics Committee of the Third People's Hospital of Hubei Province. The patients/participants provided their written informed consent to participate in this study. Written informed consent was obtained from the individual(s) for the publication of any potentially identifiable images or data included in this article.

## Author contributions

YW and QF contributed to conception and design of the study. YW organized the database. HT performed the statistical analysis. YW wrote the first draft of the manuscript. HW, DW, and HJ wrote sections of the manuscript. All authors contributed to manuscript revision, read, and approved the submitted version.
