# Corrigendum: Selective intraarterial hypothermia combined with mechanical thrombectomy for acute cerebral infarction based on microcatheter technology: a single-center, randomized, single-blind controlled study

**DOI:** 10.3389/fneur.2024.1498742

**Published:** 2024-10-09

**Authors:** Yue Wan, Hao Tian, Hui Wang, DaPeng Wang, HaiWei Jiang, Qi Fang

**Affiliations:** ^1^Department of Neurology, The First Affiliated Hospital of Suzhou University, Suzhou, Liaoning, China; ^2^Department of Neurology, Hubei Provincial Third People's Hospital, Zhongshan Hospital, Wuhan, Hubei, China

**Keywords:** infarction, endovascular therapy, hypothermia, controlled studies, oxidative stress, inflammatory response

In the published article, there was an error in the end statements as there was no **Funding statement**. The correct **Funding statement** appears below.

