# Corrigendum: Selective intraarterial hypothermia combined with mechanical thrombectomy for acute cerebral infarction based on microcatheter technology: a single-center, randomized, single-blind controlled study

**DOI:** 10.3389/fneur.2025.1620774

**Published:** 2025-05-15

**Authors:** Yue Wan, Hao Tian, Hui Wang, DaPeng Wang, HaiWei Jiang, Qi Fang

**Affiliations:** ^1^Department of Neurology, The First Affiliated Hospital of Suzhou University, Suzhou, Liaoning, China; ^2^Department of Neurology, Hubei Provincial Third People's Hospital, Zhongshan Hospital, Wuhan, Hubei, China

**Keywords:** infarction, endovascular therapy, hypothermia, controlled studies, oxidative stress, inflammatory response

In the published article, there was an error in the Funding statement. The correct Funding statement appears below.

